# Application of Gene Expression Programming (GEP) for the Prediction of Compressive Strength of Geopolymer Concrete

**DOI:** 10.3390/ma14051106

**Published:** 2021-02-26

**Authors:** Mohsin Ali Khan, Adeel Zafar, Arslan Akbar, Muhammad Faisal Javed, Amir Mosavi

**Affiliations:** 1 Department of Structural Engineering, Military College of Engineering (MCE), National University of Science and Technology (NUST), Islamabad 44000, Pakistan; moak.pg18mce@student.nust.edu.pk (M.A.K.); adeel.zafar@mce.nust.edu.pk (A.Z.); 2 Department of Architecture and Civil Engineering, City University of Hong Kong, Kowloon 999077, Hong Kong, China; 3 Department of Civil Engineering, Comsats University Islamabad, Abbottabad 22060, Pakistan; arbabfaisal@cuiatd.edu.pk; 4 Faculty of Civil Engineering, Technische Universität Dresden, 01069 Dresden, Germany; 5 School of Economics and Business, Norwegian University of Life Sciences, 1430 Ås, Norway; 6 John von Neumann Faculty of Informatics, Obuda University, 1034 Budapest, Hungary; 7 John School of the Built Environment, Oxford Brookes University, Oxford OX3 0BP, UK

**Keywords:** artificial intelligence, gene expression programming, fly ash, waste materials, geopolymer, regression analysis, building materials, sustainable construction materials, smart cities, sustainable concrete, cement

## Abstract

For the production of geopolymer concrete (GPC), fly-ash (FA) like waste material has been effectively utilized by various researchers. In this paper, the soft computing techniques known as gene expression programming (GEP) are executed to deliver an empirical equation to estimate the compressive strength $f_{c}^{\prime }$ of GPC made by employing FA. To build a model, a consistent, extensive and reliable data base is compiled through a detailed review of the published research. The compiled data set is comprised of 298 $f_{c}^{\prime }$ experimental results. The utmost dominant parameters are counted as explanatory variables, in other words, the extra water added as percent FA ($\%E_{W}$), the percentage of plasticizer (%P), the initial curing temperature (T), the age of the specimen (A), the curing duration (t), the fine aggregate to total aggregate ratio ($F/A_{G}$), the percentage of total aggregate by volume ($\;\%A_{G}$), the percent SiO_2_ solids to water ratio (% S/W) in sodium silicate (Na_2_SiO_3_) solution, the NaOH solution molarity (M), the activator or alkali to FA ratio ($A_{L}/F_{A}$), the sodium oxide (Na_2_O) to water ratio (N/W) for preparing Na_2_SiO_3_ solution, and the Na_2_SiO_3_ to NaOH ratio ($N_{s}/N_{o}$). A GEP empirical equation is proposed to estimate the $f_{c}^{\prime }$ of GPC made with FA. The accuracy, generalization, and prediction capability of the proposed model was evaluated by performing parametric analysis, applying statistical checks, and then compared with non-linear and linear regression equations.

## 1. Introduction

Fly ash (FA) is the unburned leftover residue from thermal coal plants [1]. Which is transported by gases emitted from the burning zone in the boiler. FA is collected through mechanical or electrostatic separator [2]. Annually around 375 million tons of FA is produced throughout the globe, with a disposal cost as high as $20–$40 per ton [3]. It is dumped into landfills in sub-urban areas [4]. However, dumping tons of FA exclusive of treatment sets off a malignant impact on the green environment [5]. The hazardous materials in FA like silica, alumina, and oxides such as a ferric oxide (Fe_2_O_3_) are intervening factors in water, soil, and air pollution. This ultimately leads to health issues and different geo-environmental problems [6,7]. A good waste management employment is desirable for the sustainability of a safe environment [8]. FA, if not properly disposed of, will affect the whole ecological cycle. Ultra-fine particles of FA act in the same way as poison when they reach the respiratory system. Consequently, causing physiological disorders and other related health issues like cancer, hepatic disorder, anemia, dermatitis, and gastroenteritis. FA pollutes surface and underground water which stresses aquatic life and causes skin diseases and diarrhea [7].

Concrete is used worldwide as a construction material and is classified as the second most consumable material after water [9,10]. It is reported that for every human about three tons of concrete is produced [11]. Around 25 billion tons of concrete is manufactured every year globally [12]. According to current world stats, approximately 2.6 billion tons of cement is produced per year. This will rise by 25 percent in the next 10 years [13]. However, the manufacturing of cement has an adverse effect on the environment. One ton of CO_2_ is emitted into the air to produce one ton of cement. This creates an alarming situation for the environment. Limestone is the major resource of ordinary Portland cement. Severe limestone unavailability could occur in 25–50 years [14,15]. The worldwide construction industry consumes one-third of the entire resources and is liable for 30 percent CO_2_ release globally. Thus, production of green concrete is important to reduce adverse environmental effects [16,17]. FA can be used as supplementary material in the cementitious matrix. It has been utilized by researchers to make green concrete [18,19,20,21]. FA utilization in the construction industry is a smart choice as it will reduce the usage of cement and the harmful effect of its disposal into landfills.

The utilization geopolymer concrete made of FA-like waste, is on the rise for the last two decades as lesser amounts of cement are used in geopolymer concrete (GPC) [22,23,24,25,26]. FA has been used effectively in the construction industry but its application is still limited due to the anomalous behavior of FA [27]. FA-dependent GPC is adopted extensively by builders. No method is available to estimate the mechanical properties of FA-based GPC based on a mix ratio with maximum variables. The mechanical properties of FA-based GPC critically depends on several parameters like the extra water added as percent FA ($\%E_{W}$), the percentage of plasticizer (%P), the initial curing temperature (T), the age of the specimen (A), the curing duration (t), the fine aggregate to total aggregate ratio ($F/A_{G}$), the percentage of total aggregate by volume ($\;\%A_{G}$), the percent SiO_2_ solids to water ratio (% S/W) in sodium silicate (Na_2_SiO_3_) solution, the NaOH solution molarity (M), the activator or alkali to FA ratio ($A_{L}/F_{A}$), the sodium oxide (Na_2_O) to water ratio (N/W) for preparing Na_2_SiO_3_ solution, and the Na_2_SiO_3_ to NaOH ratio ($N_{s}/N_{o}$) [13,28,29,30,31,32,33,34,35]. This creates ambiguity in the prediction properties of GPC. Moreover, a rapid spike in the use of soft computing techniques to build an empirical model has been observed recently [36,37]. Gene expression programming (GEP) is one of the popular soft computing methods utilized by various researchers in several engineering perspectives. Actual GEP is inspired by the reproduction of DNA molecules at gene level [38]. Tanyildizi et al. [39] predicted different mechanical properties of lightweight concrete subjected to elevated temperature. The author projected two different GEP models with chromosome levels equal to 30, head size 8, and number of genes equal to 4. Multiplication and addition are the two different linking functions used. The execution time of the GEP depends on the chromosome level, which dictates the size of the population. Genetic operators help in the genetic variation of the chromosomes. The chromosome that delivers the best results is forwarded to subsequent generations and the process is repeated until the achievement of an acceptable fitness.

Recently, different researchers use the GEP for the estimation of various mechanical characteristics of different types of concrete. The researchers use experimental and literature-based data for the prediction of compressive strength of sugar cane bagasse ash (SCBA) concrete via GEP [36]. Furthermore, the authors proposed a formula using GEP for estimating the axial capacity of concrete filled steel tube (CFST) with just 277 instances [37]. Furthermore, Nour et al. [40] worked with GEP algorithms for the estimation of compressive strength of CFST containing recycled aggregates.

## 2. Supervised Machine Learning Algorithms

Artificial neural networks (ANN), fuzzy logic, genetic algorithms (GA), and genetic programming (GP) use AI techniques built on natural tools. These methods have been used to resolve the problems of the pre-mix design of rubberized concrete and waste foundry sand concrete by training of the available data collected from the literature [41,42]. The configuration detection capabilities of the AI methods (support vector regression or ANN) lead to the generalization of complicated patterns. Therefore, they can be applied in the vast field of engineering [43]. By employing such approaches, the presence of an enormous sum of hidden or concealed neurons often makes it impossible to establish accurate relations between the inputs and outcomes. ANN can be exercised for estimating the mechanical properties of concrete. Recently, Getahun et al. used ANN on 66 experimental datasets to estimate the compressive strength of rice husk ash-based concrete [44]. While Mashhadban et al. predict the workability of self-compacting concrete using ANN [45]. These models give a strong correlation with no empirical expression which can be practically used. This is because of the complexity of the ANN model structure which is considered as the main obstruction in the wide-scale implementation of the ANN approach [46]. Multicollinearity is the hindrance in such methods [47]. The updated ANN technique was likewise extended to assess silica fume concrete compressive strength ($f_{cc}$) and elastic modulus ($E_{c}$) of concrete incorporates recycled aggregate. Because of the complexities of the relationship proposed, a devoted graphical interface was created for the model functional usage [48].

A strong soft computing technique, namely, genetic programming (GP), is valuable as it ignores the previous forms of established relationships for the development of the model [49,50]. An extension of GP, namely, gene expression programming (GEP), which encodes a small program and uses fixed-length linear chromosomes, was recently introduced [51]. GEP has an advantage in that a simple mathematical expression can represent the outcome that is appropriate for practicable usage of better predictive accuracy. It is currently exercised as a substitute to the common techniques of prediction [52,53,54,55,56,57,58].

Compressive strength ($f_{c}^{\prime }$) is considered as the primary factor in designing and analyzing concrete [59]. The researchers focused on the experimental route to estimate the $f_{c}^{\prime }$ of FA dependent GPC [60,61]. To save time, cost, and to sustain fly-ash and cement for future use, the development of accurate and reliable expressions is needed to relate the mix design variables and $f_{c}^{\prime }$ of GPC made with FA. A complete and thorough revision of the literature discloses that there are few empirical models for the estimation the $f_{c}^{\prime }$ of FA based GPC [41,55,58]. Though, the predictions of such empirical equations are confined to a specific dataset, for example, to the corresponding experimental study results. The prediction from such models is not viable and accurate outside the corresponding database file. Alkaroosh et al. [62] developed an empirical equation to estimate the $f_{c}^{\prime }$ of FA based GPC, based on 56 data points collected from previous research [63]. In the proposed equation, no factor was used for making the sodium silicate solution. Their equation shows a pure linear relationship between the NaOH solution molarity and $f_{c}^{\prime }$ for FA-based GPC. While other researchers reported a decrease in compressive strength by increasing the molarity of the NaOH solution [64]. To fill the research gap, the GEP approach is employed to establish a generalized and more effective empirical equation for the estimation of $f_{c}^{\prime }$ of FA-based GPC with a tolerable error. A detailed database has been developed from published research that incorporates cylindrical specimen of size 200 × 100 mm, height × diameter, and cubic specimens of size 150 mm and 100 mm. The comprehensive database accomplishment guarantees that the models are consistent and accessible for the data that is not exercised in the model’s establishment. The model’s performance is also verified by observation of the statistical errors, parametric analysis, sensitivity checks, and linear and non-linear regression methods.

## 3. Research Methodology

In this segment, methodology for the establishment of an empirical model for the compressive strength ($f_{c}^{\prime }$) of GPC made with FA has been incorporated.

### 3.1. Brief Review of Genetic Programming and Gene Expression Programming

**Figure 1:**
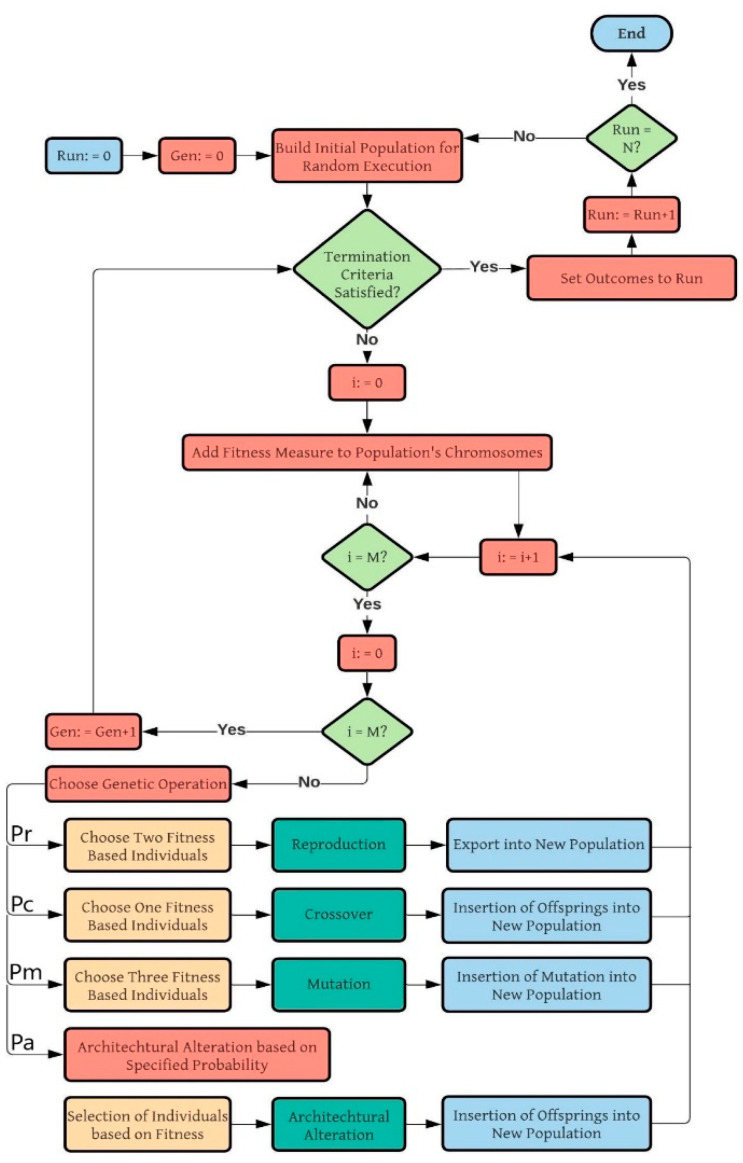
Flowchart of the genetic programming (GP) algorithm.

Koza proposed a GP method, to provide an alternate method for fixed-length binary strings (used in GAs) [65]. This method is illustrated in Figure 1 which is adapted from [37]. Five main parameters to be defined throughout the GP methodology are the collection of the terminals (the constants and the input variables), the set of primitive functions (domain-explicit functions), the fitness evaluation, the control variables (cross-over and population size, etc.) and the termination criteria followed by a result designation method [65]. The induction of non-linear parse tree-like structures makes GP an adaptable programming technique. It assumes any initial non-linearity depending on the data. A similar kind of non-linearity has been used previously [62,65]. Limitation of GP is the ignorance of the independent genome. GP uses non-linear structures that act as both the genotype and the phenotype. This makes it unlikely to produce basic and simplistic expressions. The GEP method is proposed by Ferreira, as a modified version of the GP method to overcome its discrepancies [65]. A significant alteration throughout GEP is that only the genome is transmitted towards the subsequent generation. One other noteworthy characteristic is the establishment of entities by a single chromosome composed of various genes [66]. Every gene within GEP comes in the form of fitted lengths parameters, terminal sets of constants, and the functions used are the arithmetic operations. Furthermore, in genetic code operators, there is a stabilized interaction amongst both the associated function and the chromosome symbol. The necessary information for the development of an empirical model is registered into the chromosomes and to infer this data a novel program, in other words, karva is established.

**Figure 2:**
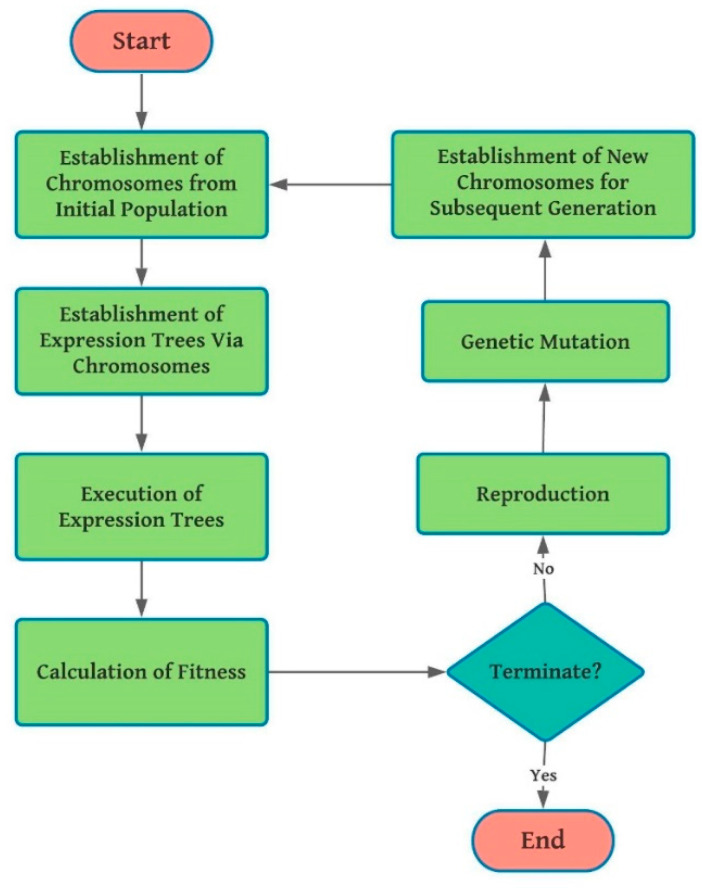
Gene expression programming (GEP) algorithm flowchart.

The phases covered in the process of GEP are illustrated in Figure 2 which is adapted from [37]. The method starts with the arbitrary formation of fixed-size chromosomes for all individuals; which are subsequently converted into expression trees (ET) and for each individual, the fitness strength is estimated. For several creations, the replication lasts with new individuals until the accomplishment of fine results. Genetic functions like crossover, reproduction, and mutation are implemented for population alteration.

### 3.2. Data Collection

Compressive strength ($f_{c}^{\prime })$ is the main factor in analysis and design of concrete structure. To save time, cost, and to sustain the use of FA in the construction industry, the development of accurate and reliable expression is needed that can relate the mix proportion and $f_{c}^{\prime }$ of GPC made with FA.

A detailed database for the $f_{c}^{\prime }$ of FA-based GPC, was compiled from previously published, experimental researches [60,61,63,67,68,69,70,71,72,73,74,75,76,77,78,79,80,81,82,83,84,85,86,87,88,89,90,91,92,93,94,95,96,97,98,99]. The database comprises of total 298 samples which include 101 cylindrical specimens of size 200 mm × 100 mm, height × diameter, 166, and 31 cube specimens of size 150 mm and 100 mm, respectively. $f_{c}^{\prime }$ of cube and cylindrical specimens depends on the length to diameter (L/D) ratio [100,101]. The $f_{c}^{\prime }$ of 100 mm cubes are 5% greater than 150 mm cubes. While $f_{c}^{\prime }$ of 150 mm cubes are 20% greater than cylindrical specimens of size 100 mm × 200 mm. With the increase of the volume of the specimen, the number of voids also increases, so, the specimen with smaller dimension will have lesser $f_{c}^{\prime }$ than the larger dimension specimen. Furthermore, the stress is inversely related to the cross-sectional area of the specimen. The one with smaller cross-sectional area will have higher stresses, which means high internal resistance to failure. Table 1 displays the normalization of the compressive strength of various types of specimens considered in this study. The comprehensive database guarantees the model reliability and accessibility for the data that is not exercised in the development of the empirical model.

**Figure 3:**
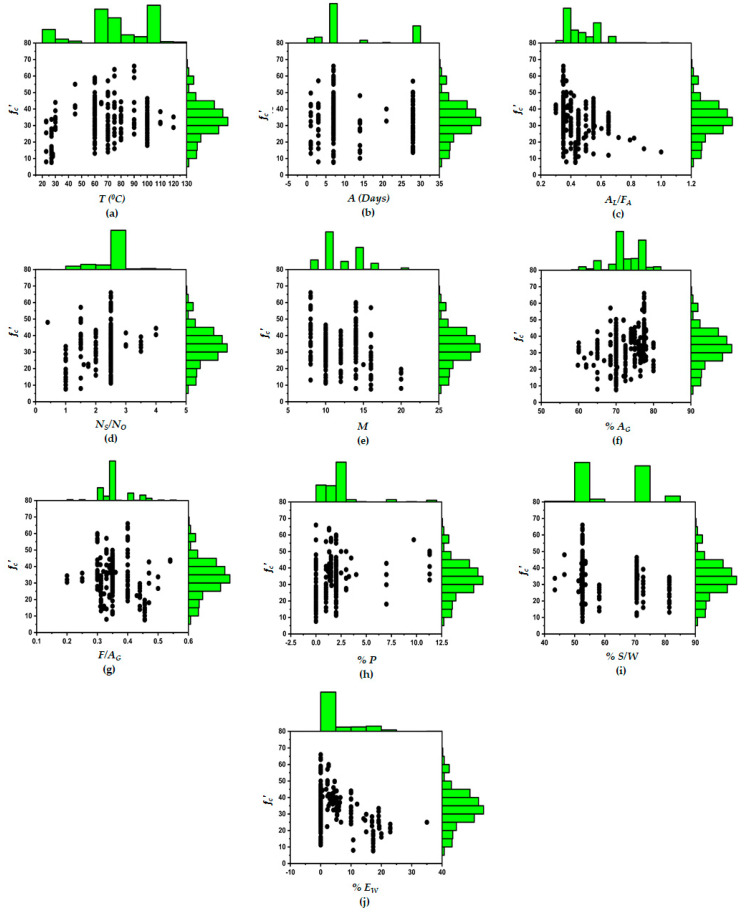
Marginal histogram of explanatory parameters against output variables. (**a**) Temperature for curing of specimen *(T^0^C),* (**b**) Age of specimen *(A),* (**c**) Alkali to fly-ash ratio *(A_L_/F_A_)*, (**d**) Sodium silicate to sodium hydroxide ratio *(N_S_/N_O_)*, (**e**) Molarity of NaOH solution *(M),* (**f**) Percentage of total aggregate by volume *(A_G_),* (**g**) Fine aggregate to total aggregate ratio *(F/A_G_)*, (**h**) Percentage of superplasticizer *(% P)*, (**i**) Percentage of SiO_2_ solids to water ratio *(% S/W)*, (**j**) Percentage of extra water added *(% E_W_)*.

The composed database covers data about the explanatory parameters, namely, the extra water added as percent FA ($\%E_{W}$), the percentage of plasticizer (%P), the age of the specimen (A), the curing duration (t), the fine aggregate to the total aggregate ratio ($F/A_{G}$), the percentage of total aggregate by volume ($\;\%A_{G}$), the percent SiO_2_ solids to water ratio (% S/W) in sodium silicate (Na_2_SiO_3_) solution, the NaOH solution molarity (M), the activator or alkali to FA ratio ($A_{L}/F_{A}$), and the Na_2_SiO_3_ to NaOH ratio ($N_{s}/N_{o}$) for the response of compressive strength. All the samples collected for the mentioned parameters are heat cured initially for 24 h at different temperatures. The $f_{c}^{\prime }$ increases with curing time but researchers reported that the rate of increment in the $f_{c}^{\prime }$ of FA-based GPC is rapid until 24 h [63]. The early strength of GPC is higher due to the geopolymerization process and limited literature is available for longer curing duration. Van Jaarsveld et al. [102] described that for longer than 24 h curing time, the $f_{c}^{\prime }$ is not increased. Every model performance depends on the distribution of explanatory parameters [103]. The marginal histograms of all ten input parameters used in this study are shown in Figure 3, which dictates that all 10 explanatory parameters selected are distributed through its range for the compressive strength. The bar charts added above and to the right of the main plot add more information to the data. Along with the distribution of the input variables, it also shows the distribution of the compressive strength. Every explanatory variable has a strong impact on the variation of the compressive strength of FA-based GPC.

To conduct the generalized study, both cubes and cylindrical specimens are counted to construct a database. The output and input variables’ ranges, along with their mean values are presented in Table 2. For the achievement of reliable and consistent predictions of the compressive strength, it is endorsed to utilize the suggested model with the ranges provided.

It should be noted that, for the evaluation of the validity, reliability, and consistency of the database, multiple trials were conducted. Datasets that diverged considerably from the global norm (about 20%) were not included in the model’s creation and performance evaluation. To establish an empirical model, 298 datasets for the prediction of compressive strength were used. In this research, the data points were arbitrarily divided into two statistically consistent sets known as the training and validation sets [37]. Furthermore, 70% (208 data points) of the total data are assigned to the training set and 30% (90 data points) to the validation set [37]. The training set was employed for training the empirical model known as gene progression, whereas validation data points were utilized for the justification and calibration of the established model’s generalization capability as suggested in the literature [57].

### 3.3. Model Development and Evaluation Criteria

For the development of the model, the first step is the selection of input parameters that can influence the FA-based GPC’s properties. Influential parameters that effect the compressive strength ($f_{c}^{\prime }$) of GPC made with FA were selected for the generalized model development. The detailed study is carried out and the performance of several initial runs is computed. Hence, the FA-based GPC’s compressive strength is taken into account as the function of Equation (1).

(1) $$f_{c}^{\prime }=f\left(T,\;A,\;M,\%\frac{S}{W},\;\frac{A_{L}}{F_{A}},\frac{N_{S}}{N_{O}}A_{G},\frac{F}{A_{G}},\;\%P,\%E_{W}\right)$$

Chromosomes, genes, and expression trees (ETs) perform a central role in the development of the GEP model. The program’s running duration is regulated through the size of the population (chromosome number). The chromosome is comprised of genes that are used for encoding of the subexpression trees (sub-ETs). Considering the predictive model complexity, the stages counted as population size were 150. The model’s architectural structures rest on the gene number and head-size with the latter dictating the difficulty of every term and the latter deciding the sum of the model’s sub-ETs. Thus, population size 150, genes 3, and head size 10 is considered for the development of the model. The chromosomes are subjected to genetic variation through genetic operators. In mutation, the component of the gene’s tail or head is randomly selected and replaced with a randomly selected component of the terminal or function set. The transposition function involves the transposition of the sequences inside the chromosomes, in other words, root insertion sequence (RIS) and insertion sequence (IS). After all, the recombination combines and splits up 2 chromosomes in order to substitute their elements. For creating the fair empirical model, the adjusted settings recommended in earlier literature were used [41]. To execute the GEP algorithm, GeneXproTool was used. Table 3 illustrates the adjusted setting of the hyperparameters utilized in the formation of the GEP empirical equation.

A correlation coefficient (R) is mostly applied to measure model performance. However, it cannot be merely studied as the sign of model predictive accuracy as it is insensitive towards division and multiplication of outcomes to a constant [104]. For that reason, in this research the mean absolute error (MAE), the root means square error (RMSE), the relative root mean squared error (RRMSE), and the relative squared error (RSE) are also considered. Moreover, the model performance evaluation performance index (ρ) is recommended, as it covered the function of both the R and RRMSE [103]. The equations of error functions used in this study are provided as Equations (2)–(7):

(2) $$RMSE=\sqrt{\frac{\sum_{i=1}^{n}{\left(e_{i}-m_{i}\right)}^{2}}{n}}$$

(3) $$MAE=\frac{\sum_{i=1}^{n}\left|e_{i}-m_{i}\right|}{n}$$

(4) $$RSE=\frac{\sum_{i=1}^{n}{\left(m_{i\;}-e_{i}\right)}^{2}}{\sum_{i=1}^{n}{\left(\bar{e}-e_{i}\right)}^{2}}$$

(5) $$RRMSE=\frac{1}{\left|\bar{e}\right|}\sqrt{\frac{\sum_{i=1}^{n}{\left(e_{i}-m_{i}\right)}^{2}}{n}}$$

(6) $$R=\frac{\sum_{i=1}^{n}(e_{i}-{\bar{e}}_{i})\left(m_{i}-{\bar{m}}_{i}\right)}{\sqrt{\sum_{i=1}^{n}{\left(e_{i}-{\bar{e}}_{i}\right)}^{2}\sum_{i=1}^{n}{\left(m_{i}-{\bar{m}}_{i}\right)}^{2}}}$$

(7) $$\rho =\frac{RMSE}{1+R}$$

where $m_{i}$ and $e_{i}$ are the $i^{th}$ model outcome value and experimental value, respectively. While ${\bar{m}}_{i}$ and ${\bar{e}}_{i}$ are the model’s outcome average value and experimental average value, respectively. Additionally, *n* denotes the overall data points. High R-value and low RMSE, MAE, RSE, and RRMSE shows a best-calibrated model. It is suggested that for a deep correlation between measured and predicted values, the R-value should be greater than 0.8 (as for ideal model R = 1) [105]. The (ρ) value near to zero replicates better model performance.

## 4. Results and Discussion

**Figure 4:**
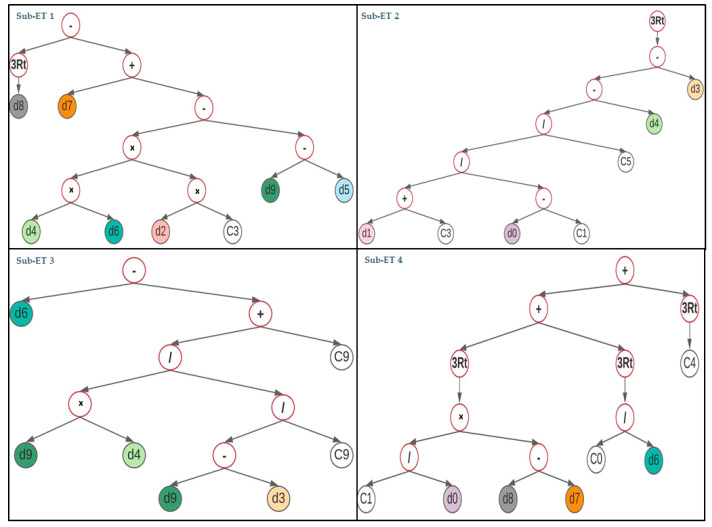
GEP model expression trees (ET_S_) for compressive strength *f_c_^′^*.

The GEP algorithm’s output for the compressive strength ($f_{c}^{\prime })$ model as an expression tree is shown in Figure 4. The empirical relationship was obtained by decoding these expression trees (ETs) that encompass five arithmetical operations, namely, +, −, ×, / and ∛.

GEP ETs use the indicators to express the explanatory variables. The corresponding symbols and description of each indicator are provided in Table 4.

### 4.1. Compressive Strength Formulation for FA Based Geopolymer Concrete

Equation (8) is the simplified equation that is presented to estimate the compressive strength, $f_{c}^{\prime }$, for GPC made with FA in MPa. It is comprised of four variables namely *A*, *B*, *C*, and *D* represented as Equations (9)–(12) and have been translated from the sub-ETs 1, 2, 3, and 4, respectively, as illustrated in Figure 4.

(8) $$f_{c}^{\prime }\left(MPa\right)=A\times B\times C\times D$$

where;

(9) $$A=\sqrt[{3}]{\frac{S}{W}\%}-P\%+\left(M\times \frac{F}{A_{G}}\times \frac{A_{L}}{F_{A}}\times 6.61\right)+E_{W}\%-A_{G}\%$$

(10) $$B=-\sqrt[{3}]{\frac{A+80}{0.083\left(T-17.87\right)}+M+\frac{N_{S}}{N_{O}}}$$

(11) $$C=\frac{F}{A_{G}}-\left(E_{W}\%\times M-\frac{0.0003}{\frac{N_{S}}{N_{O}}-E_{W}\%}\right)-0.0003$$

(12) $$D=\sqrt[{3}]{\frac{\left(P\%-\frac{S}{W}\%\right)1.16}{T}}+\sqrt[{3}]{\frac{0.17}{\frac{F}{A_{G}}}}+0.77$$

**Figure 5:**
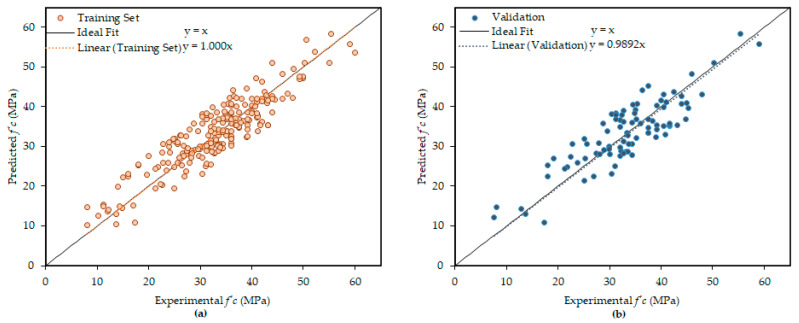
Experimental and predicted compressive strength values comparison: (**a**) training set values and (**b**) validation set values.

Figure 5 represents the comparison of regression lines between experimental and model outcomes for both the training samples and validation samples. A strong correlation can clearly be seen which is represented via slopes of the regression lines, namely, 1.000 and 0.9892, for the train and validation samples, respectively.

### 4.2. Sensitivity and Parametric Analysis

**Figure 6:**
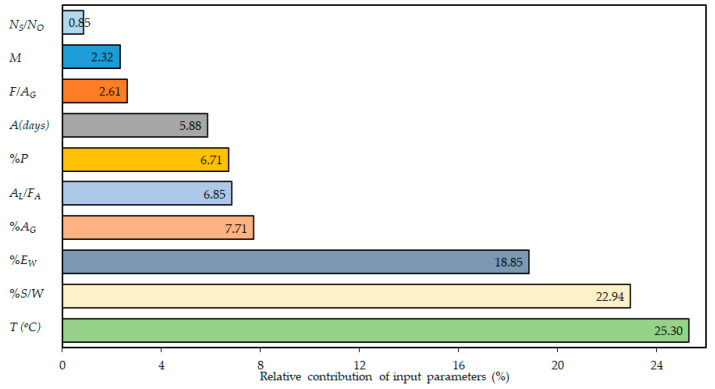
Percent relative contribution of input parameter.

Sensitivity analysis (SA) is performed to investigate the relative contribution of input variables that are exercised to estimate the compressive strength $f_{c}^{\prime }$ of GPC made with FA, using Equations (13) and (14). SA defines the dependency of the outcome on the input variable.

(13) $$N_{i}=f_{max}\left(x_{i}\right)-f_{min}\left(x_{i}\right)$$

(14) $$SA=\frac{N_{i}}{\sum_{n}^{j=1}N_{j}}$$

where $x_{i}$ represents the $i^{th}$ input variable. $f_{max}\left(x_{i}\right)$ and $f_{min}\left(x_{i}\right)$ represent the maximum and minimum values of outcome, respectively, that depends on its $i^{th}$ input dominion, where other input variables are maintained at a constant average value. The difference between $f_{max}\left(x_{i}\right)$ and $f_{min}\left(x_{i}\right)$ gives the range $N_{i}$ of the $i^{th}$ input variable. The sensitivity and parametric study were both conducted for the training data set, as both the training and validation data sets are consistent [41,105]. Results of sensitivity analysis are presented in Figure 6. The figure clarifies that, from a material engineering perspective, the involvement of the explanatory parameters to the $f_{c}^{\prime }$ of GPC made with FA are similar.

**Figure 7:**
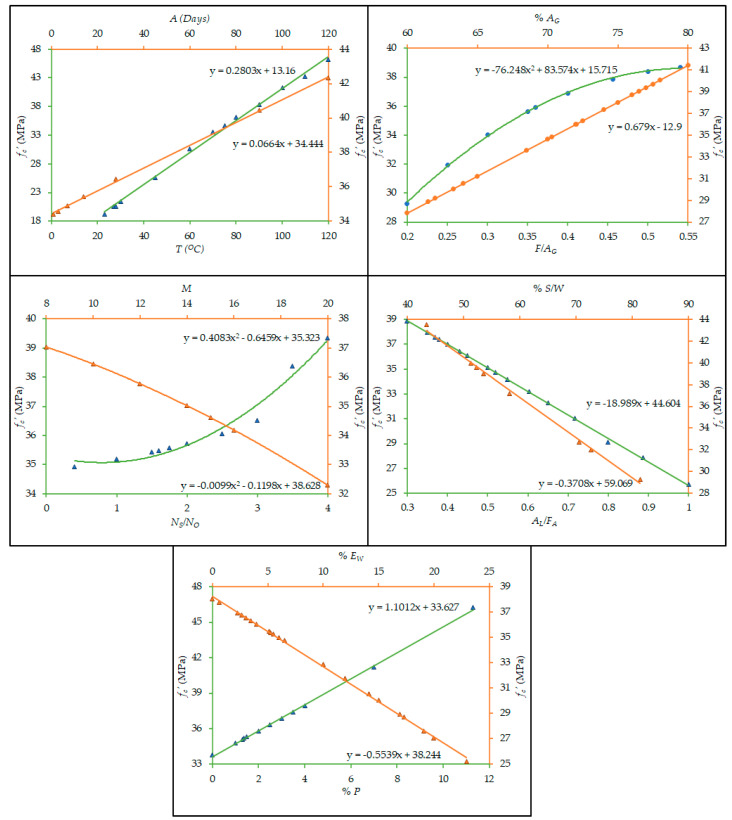
Influence of input parameters variation on the compressive strength.

Besides, the effectiveness of most influential input variables in the projection of the compressive strength of FA-dependent GPC is obtained by performing parametric analysis. Changes in compressive strength were recorded only by changing the value of one variable from maximum to minimum and other inputs were maintained at average values. Figure 7 illustrates the GEP model’s parametric analysis results.

It is known that the temperature for curing of the samples is the prompting parameter in controlling the compressive strength ($f_{c}^{\prime })$ of GPC made with FA. Its relative contribution is 25.3% as depicted in Figure 6. Figure 7 shows that the $f_{c}^{\prime }$ increases at various rates with the increase of T, A,$\;\%A_{G}$, $\left(F/A_{G}\right)$, $\left(N_{s}/N_{o}\right)$, and %P, but decreases with $\%E_{W}$, $\left(A_{L}/F_{A}\right)$, M, and (%S/W).

Hydrates and silicates are released by the alkali-activating solution that helps in the formation of the polymeric structure of alumina silicates. Extra heat is needed for speeding up the reaction process and to improve the mechanical characteristics of GPC. Figure 7 shows that the $f_{c}^{\prime }$ increases with the increase in the curing temperature up to 100 °C. At higher curing temperature the moisture from the concrete is lost, even if sealed properly. Analogous results have also been witnessed in earlier literature [64]. The decrease in the rate of increment of $f_{c}^{\prime }$ of GPC after 240 days, is due to the decrease in the number of unreacted particles. Wardhono et al. [73] presented scanning electron microscopy (SEM) images, which show that gel fills up the voids after 240 days leading to the formation of compacted and semi-homogenous microstructure. Furthermore, it can be depicted from Figure 7 that the $f_{c}^{\prime }$ increases with an increase in the amount of total aggregate, however, the total aggregate relates to the ratio between fine aggregate to total aggregate content.

Alkali to FA ratio is linked to the ratio between sodium silicate to sodium hydroxide, and the molarity of NaOH. The increase in the $f_{c}^{\prime }$ is greatly altered with the amount of sodium silicate that transforms the microstructure of GPC. In the development of the sodium silicate solution, the ratio between percentage silica to water needs to be higher. The higher the sodium silicate content, the greater the compressive strength will be. The lower ratio of alkali to fly ash in combination with higher sodium silicate to sodium hydroxide ratio, and lower molarity of NaOH solution results in higher compressive strength. However, the amount of NaOH solution must remain enough to complete the process of dissolution of the geopolymer. Similar findings have also been observed in a previous study [74].

In GPC, the total water content is the addition of the water content required in preparing the solution of sodium silicate and sodium hydroxide and the addition of extra water needed. To prevent cracking and to achieve a practical GPC, it is necessary to consider the addition of extra water and plasticizer [90]. The addition of extra water or plasticizer as a percent FA contributes 18.85% and 6.71% respectively to the $f_{c}^{\prime }$ in comparison with other input factors. $f_{c}^{\prime }$ of GPC increases with the increase in plasticizer and decreases with the addition of extra water as evident from Figure 7, as the addition of extra water beyond certain limits leads to bleeding and segregation in fresh concrete mix.

Figure 7 is in line with the previous studies of other researchers [74,90]. The results of parametric analytics for the proposed GEP model correctly encompasses the influence of input variables to estimate the $f_{c}^{\prime }$ of FA-based GPC.

### 4.3. Performance Evaluation of GEP Models

According to the previous study, to achieve a reliable GEP equation, the ratio between the number of data points in the database to the number of input parameters should be at least equal to three [103]. While in this study a higher value of 30 has been used. Table 5 represents the statistical analysis for validation sets and training sets of the GEP model. These results illustrate the effectiveness of training models and the strong correlation between experimental and predicted outcomes with minimal error. The RMSE, MAE, and RSE for the training set of the GEP model are 5.971, 5.832, and 0.325, respectively, and are calculated as 2.643, 2.057, and 0.0675 from the validation samples. The statistical measure of the training and validation set are similar, which indicates the higher generalization capability of the model. Thus, the developed model can predict accurate and reliable outcomes for the new data. Table 5 witnesses ρ approach zero (as ideal cases equal zero).

**Figure 8:**
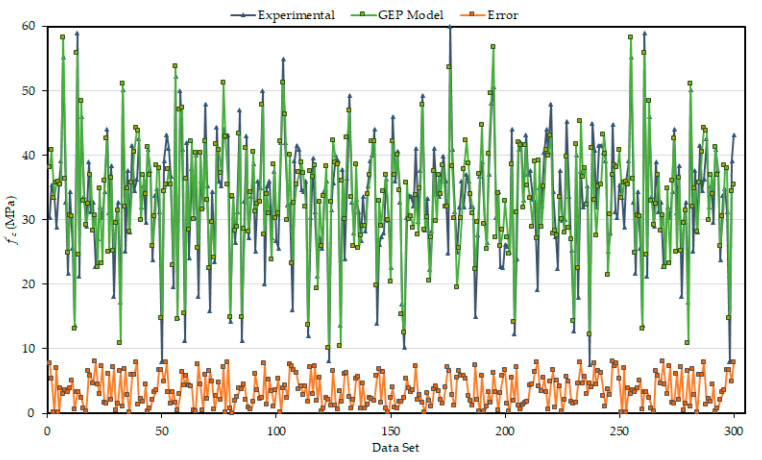
Absolute error representation of experimental and predicted outcomes.

Figure 8 illustrates the absolute error of both the experimental and predicted model outcomes, which gives an overall idea of the maximum percentage of error. The average percent error and maximum percent error were calculated as 6.47% and 8.32% respectively, which confirms that the experimental and model outcomes are similar. Furthermore, the occurrence frequency for the maximum error is comparatively smaller. Almost 90% of model predictive outcomes for validation set have the error below 10%, while the average percent error is less than 5.56%. This verifies the accuracy and generalization of the developed GEP equation.

For external validation and testing of the proposed GEP model, various statistical error tests were also employed. The literature discloses a suggested criterion that the slope (inclination) of any of the regression lines ($k\;or\;k^{\prime }$) traversing the origin should be approximately equal to 1 [106]. The slope of regression lines is 1.001 and 0.995 as shown in Table 6. It shows greater accurateness and correlation. Moreover, the researchers proposed that the squared correlation coefficient (passing by origin) among the predicted outcome and experimental results ($R_{o}^{2}$) or among the experimental and predicted outcome (${R_{o}^{\prime }}^{2}$) should approach 1 [107]. Table 6 summarizes these checks and was applied to the developed GEP equation. The results of these external validations replicate that the proposed GEP model is valid. Thus, the proposed model is not only a correlation but also has predictive capacity.

### 4.4. Comparison of GEP and Regression Models

No GEP model has been identified from the literature that would estimate the compressive strength ($f_{c}^{\prime }$) of GPC made with FA and that considers the influencing input variables used in this research. So, it is necessary to establish linear and non-linear regression models, on the same data points, for the prediction of the $f_{c}^{\prime }$ of FA-based GPC, the results are then judged against GEP Equation (8).

Equations (15) and (16) show the empirical expressions for the prediction of $f_{c}^{\prime }$ founded on linear and nonlinear regression study respectively.

(15) $$\begin{array}{l} f_{c}^{\prime }=12.81+0.226T+0.0376A-26.86\frac{A_{L}}{F_{A}}+1.1296\frac{N_{S}}{N_{o}}-0.3935M+0.6412A_{G}\%\\ -0.4075\frac{F}{A_{G}}+1.256P\%-0.452\frac{S}{W}\%-0.7125E_{W}\% \end{array}$$

(16) $$\begin{array}{l} f_{c}^{\prime }=-7.636+1.182T^{0.6809}+0.3446A^{0.634}-25.80{\left(\frac{A_{L}}{F_{A}}\right)}^{2.91}+1.779{\left(\frac{N_{S}}{N_{o}}\right)}^{0.438}\\ -0.00895M^{2.24}+0.7605{\left(A_{G}\%\right)}^{0.932}-0.37099{\left(\frac{F}{A_{G}}\right)}^{1.064}+2.259{\left(P\%\right)}^{0.7203}\\ -0.0804{\left(\frac{S}{W}\%\right)}^{1.345}-0.2654{\left(E_{W}\%\right)}^{1.316} \end{array}$$

**Figure 9:**
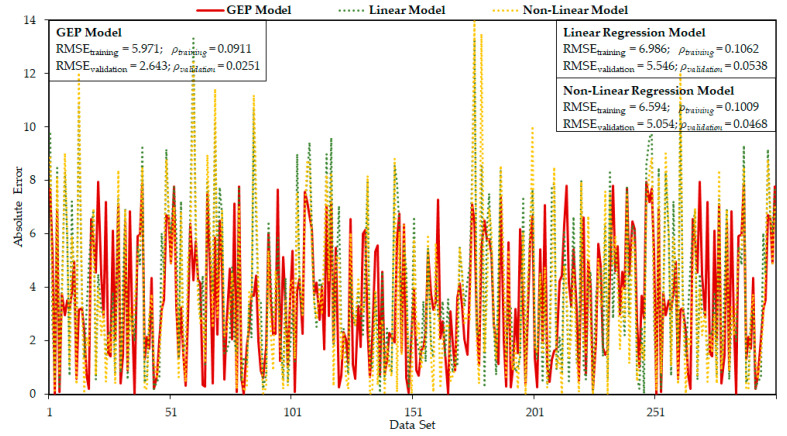
Comparison of *f_c_^′^* of proposed GEP, linear regression, and no-linear regression models.

The absolute error of predicted results by all three equations are compared in Figure 9. The statistical indicators like RMSE, MAE, RSE, RRMSE%, R, and ρ for GEP model, linear and no-linear regression model are listed in Table 5. The ρ and RMSE of the established GEP equation are calculated as the least of all three models, for both the training and validation data points. The values of RMSE_training_ and $\rho_{training}$ are 14.5% and 14% lower than the linear regression model, respectively. In the test stage, the proposed GEP model gives better performance than the non-linear regression model. $\rho_{testing}$ of the two models varies by 44%. Furthermore, Figure 9 shows that linear and non-linear regression equations failed in efficiently capturing a high $f_{c}^{\prime }$, which limits the application of the regression models.

These observations shows that the GEP model performed better than the linear and non-linear regression equations, for the same input variables. The regression methods have certain disadvantages as in they use some predefined expressions and pre-assume the residual’s normality [105]. Whereas modeling based on GEP implies that the model efficiently picks up the non-linear relationship between the dependent and independent parameters, with a higher generalization capacity and considerably decreases the error values in comparison with the regression models.

## 5. Recommendations for Future Study

Fly-ash (FA)- based geopolymer concrete (GPC) has a great potential to be used in the construction industry, as a replacement of ordinary Portland cement (OPC) concrete. The data set used in this paper is limited to 298 samples. In fact, proper testing must be carried out by varying maximum explanatory variables for a more efficient predictive model. Although, this paper considers a wide range comprehensive data base consisting of ten explanatory parameters for modelling the compressive strength of geopolymer concrete made with wasted fly-ash.

Moreover, study of other mechanical characteristics of fly-ash based GPC like tensile strength, elastic modulus, poison ratio, and flexural strength, is highly necessary; at normal temperature as well as at elevated temperature. A new data base is also needed for the durability study of fly-ash-based GPC. Furthermore, it is recommended to predict the stated mechanical properties of fly-ash-based GPC via different artificial intelligence (AI) techniques, such as fuzzy logic, adaptive fuzzy interface system (ANFIS), response surface methodology (RSM), support vector machine (SVM) analysis, random forest regression (RFR), decision tree (DT), artificial neural network (ANN), recurrent neural network (RNN), convolutional neural network (CNN), M5P tree and restricted Boltzmann machine (RBM), et cetera. Furthermore, an extensive study related to the interaction of geopolymer concrete and reinforcing steel is needed. It would also be worthwhile formalizing the different mechanical properties of fiber reinforced geopolymer concrete.

Normally it is considered that the production cost of GPC is greater than OPC concrete. It can be reduced by the use of different types of waste materials such as sand replacement that are rich in alumina silicates; like the use of locally available waste foundry sand, glass waste, and marble wastes, et cetera. The authors replaced fine aggregates with waste foundry sand in GPC. They reported that the initial production cost of M50 grade GPC is 11% lower than OPC concrete [108]. However, the M30 grades of GPC and OPC concrete have almost similar of production costs [108]. Environmental safety delivered by GPC production from waste materials is worthwhile as it reduces the carbon-dioxide emission from the manufacture of cement and adds a carbon credit to the economy of the country as well. Comparing the overall cost, including the maintenance and durability, the cost of GPC is similar to OPC concrete as the geopolymer concrete is much more durable and resistive to chemical attacks than OPC concrete [109]. The authors immersed GPC and OPC concrete in a magnesium sulfate solution for 45 days and reported that the reduction of compressive strength of GPC is 13% lower than OPC concrete [109]. Additionally, the immersion for the same duration in a sulfuric acid solution resulted in 8% lower reduction of compressive strength of GPC as compared to OPC concrete [109].

## 6. Conclusions

This research utilizes the gene expression programming technique (GEP) to establish an expression for the estimation of the compressive strength, $f_{c}^{\prime }$, of geopolymer concrete (GPC) made with fly-ash. The projected GEP model is empirical and is built on the broadly distributed database, consisting of different parameters, that comes from the published literature. For the prediction of the $f_{c}^{\prime }$ of fly-ash-based GPC, highly prominent and influential parameters are considered as explanatory variables. The predicted model results satisfy the experimental results. From the parametric analysis, it has been shown that the projected model successfully encompasses the impact of the input parameters to predict the exact pattern of fly-ash-based GPC. The accurateness of the projected models is verified by the examination and assessment of statistical checks MAE, RSE, R, and RMSE and fitness functions (ρ) for training and validation samples. Furthermore, the model correctly meets the appropriate requirements considered for external validation. The comparison of the proposed model with the simple linear and non-linear equations shows that the GEP model possesses a higher generality and predictive capability and is appropriate to practice in the preliminary design of fly-ash-based GPC. Furthermore, before adding fly-ash as a geopolymer binder, it is suggested to perform a leachate analysis. The projected models can provide a detailed and practical foundation for increasing the use of toxic fly-ash for construction practices, instead of disposal in landfill sites. This would lead to effective and sustainable construction as green concrete is made by the incorporation of waste fly-ash that reduces the consumption of energy, emissions of greenhouse gases, disposal, and construction costs.

## Figures and Tables

**Table 1:** Type of specimens and compressive strength normalization factor.

Type of Specimens	Normalized Factor
Cylindrical (200 mm × 100 mm)	1
Cubic (150 mm)	0.8
Cubic (100 mm)	0.95 × 0.8

**Table 2:** Range and average of explanatory and response parameter.

Parameters	Minimum Value	Maximum Value	Mean Value
**Explanatory parameters**			
*T* (°C)	23	120	71.57
*A* (days)	1	540	20.87
*A/F*	0.3	1	0.4545
*N_S_/N_O_*	0.4	4	2.275
*M*	8	20	11.68
*A_G_* (%)	60	80	72
*F/A_G_*	0.2	0.5	0.3568
*P* (%)	0	11.3	1.998
*S/W* (%)	43.4	81.4	61.68
*E_W_* (%)	0	35	3.889
**Response**			
*f_c_^′^* (MPa)	8.2	63	37

**Table 3:** Adjusted Setting of parameters for the GEP model.

Parameters	$\mathrm{Adjusted}\;\mathrm{Setting}\;\mathrm{for}\;f_{c}^{\prime }$
**General**	
Population chromosomes	150
Genes	4
Head size	10
Linking function	Multiplication
Function Set	$-,+,\;/,\times ,\sqrt[{3}]{\;}$
**Arithmetical Constants**	
Constant per gene	10
Data type	Floating data
Upper Bound	10
Lower bound	–10
**Genetic operators**	
Mutation rate	0.00138
Inversion rate	0.00546
IS transportation rate	0.00546
RIS transportation rate	0.00546
One-point recommendation rate	0.00277
Two-point recommendation rate	0.00277
Gene recombination rate	0.00755
Gene transportation rate	0.00277

**Table 4:** Indicators of GEP expression tree.

Indicator in Expression Tree	Description	Symbol
$d_{o}$	The temperature for curing in degrees Celsius	T
$d_{1}$	The age of the sample	A
$d_{2}$	Ratio of alkali or activator to the fly-ash	$A_{L}/F_{A}$
$d_{3}$	Ratio of Na_2_SiO_3_ to NaOH	$N_{s}/N_{o}$
$d_{4}$	NaOH solution molarity	M
$d_{5}$	Percentage of total aggregate by volume	$\%\;A_{G}$
$d_{6}$	Ratio of fine aggregate to total aggregate	$F/A_{G}$
$d_{7}$	Plasticizer as percent fly-ash	% P
$d_{8}$	Percentage of SiO_2_ solids to water ratio	%S/W
$d_{9}$	Extra water addition as percent fly ash	$\%\;E_{W}$

**Table 5:** Statistical analysis of GEP, linear, and non-linear regression models.

Model	RMSE		MAE		RSE		RRMSE (%)		R		ρ	
	T_R_^1^	V_DN_^2^	T_R_	V_DN_	T_R_	V_DN_	T_R_	V_DN_	T_R_	V_DN_	T_R_	V_DN_
GEP	5.971	2.643	5.823	2.057	0.325	0.0675	16.949	4.949	0.8586	0.9643	0.0911	0.02519
Linear	6.986	5.546	6.543	4.967	0.589	0.304	19.20	10.21	0.8074	0.8976	0.1062	0.05382
Non-Linear	6.593	5.054	6.053	4.875	0.497	0.298	18.53	9.021	0.8357	0.9247	0.1009	0.04687

^1^ T_R_ symbolizes training sample; ^2^ V_DN_ symbolizes validation samples.

**Table 6:** External validation of the GEP model using arithmetical parameter.

Expression	Requirement	$\mathrm{GEP}\;\mathrm{Model},\;f_{c}^{\prime }$
$k=\frac{\sum_{i=1}^{n}\left(e_{i}\times m_{i}\right)}{\sum_{i=1}^{n}\left(e_{i}^{2}\right)}$	0.85<k<1.15	1.001
$k^{\prime }=\frac{\sum_{i=1}^{n}\left(e_{i}\times m_{i}\right)}{\sum_{i=1}^{n}(m_{i}^{2})}$	$0.85<k^{\prime }<1.15$	0.995
$R_{o}^{2}=1-\frac{\sum_{i=1}^{n}{\left(m_{i}-e_{i}^{o}\right)}^{2}}{\sum_{i=1}^{n}{\left(m_{i}-{\bar{m}}_{i}^{o}\right)}^{2}},\;e_{i}^{o}=k\times m_{i}$	$R_{o}^{2}≅1$	0.9998
${R_{o}^{\prime }}^{2}=1-\frac{\sum_{i=1}^{n}{\left(e_{i}-m_{i}^{o}\right)}^{2}}{\sum_{i=1}^{n}{\left(e_{i}-{\bar{e}}_{i}^{o}\right)}^{2}},\;m_{i}^{o}=k^{\prime }\times e_{i}$	${R_{o}^{\prime }}^{2}≅1$	0.9849

## Data Availability

The data presented in this study are available on reasonable request from the corresponding author.

## References

[1] Aprianti E. (2017). A huge number of artificial waste material can be supplementary cementitious material (SCM) for concrete production—A review part II. J. Clean. Prod..

[2] Akbar A., Farooq F., Shafique M., Aslam F., Alyousef R., Alabduljabbar H. (2021). Sugarcane bagasse ash-based engineered geopolymer mortar incorporating propylene fibers. J. Build. Eng..

[3] Dwivedi A., Jain M.K. (2014). Fly ash—waste management and overview: A Review. Recent Res. Sci. Technol..

[4] Rafieizonooz M., Mirza J., Salim M.R., Hussin M.W., Khankhaje E. (2016). Investigation of coal bottom ash and fly ash in concrete as replacement for sand and cement. Constr. Build. Mater..

[5] Abdulkareem O.A., Mustafa Al Bakri A.M., Kamarudin H., Khairul Nizar I., Saif A.A. (2014). Effects of elevated temperatures on the thermal behavior and mechanical performance of fly ash geopolymer paste, mortar and lightweight concrete. Constr. Build. Mater..

[6] Nadesan M.S., Dinakar P. (2017). Mix design and properties of fly ash waste lightweight aggregates in structural lightweight concrete. Case Stud. Constr. Mater..

[7] Ghazali N., Muthusamy K., Wan Ahmad S. Utilization of Fly Ash in Construction. Proceedings of the IOP Conference Series: Materials Science and Engineering.

[8] Nordin N., Abdullah M.M.A.B., Tahir M.F.M., Sandu A.V., Hussin K. (2016). Utilization of fly ash waste as construction material. Int. J. Conserv. Sci..

[9] Farooq F., Akbar A., Khushnood R.A., Muhammad W.L.B., Rehman S.K.U., Javed M.F. (2020). Experimental investigation of hybrid carbon nanotubes and graphite nanoplatelets on rheology, shrinkage, mechanical, and microstructure of SCCM. Materials (Basel).

[10] Liew K.M., Akbar A. (2020). The recent progress of recycled steel fiber reinforced concrete. Constr. Build. Mater..

[11] Watts J. (2019). Concrete: The most destructive material on Earth. Guardian..

[12] Mehta P.K. (2002). Greening of the Concrete Industry for Sustainable Development. Concr. Int..

[13] Wongsa A., Siriwattanakarn A., Nuaklong P., Sata V., Sukontasukkul P., Chindaprasirt P. (2020). Use of recycled aggregates in pressed fly ash geopolymer concrete. Environ. Prog. Sustain. Energy.

[14] Sumanth Kumar B., Sen A., Rama Seshu D. (2020). Shear Strength of Fly Ash and GGBS Based Geopolymer Concrete. Lecture Notes in Civil Engineering.

[15] Farooq F., Rahman S.K.U., Akbar A., Khushnood R.A., Javed M.F., alyousef R., Alabduljabbar H., Aslam F. (2020). A comparative study on performance evaluation of hybrid GNPs/CNTs in conventional and self-compacting mortar. Alex. Eng. J..

[16] Li H., Deng Q., Zhang J., Xia B., Skitmore M. (2019). Assessing the life cycle CO2 emissions of reinforced concrete structures: Four cases from China. J. Clean. Prod..

[17] Akbar A., Liew K.M. (2020). Influence of elevated temperature on the microstructure and mechanical performance of cement composites reinforced with recycled carbon fibers. Compos. Part B Eng..

[18] Ok Y.S., Yang J.E., Zhang Y.S., Kim S.J., Chung D.Y. (2007). Heavy metal adsorption by a formulated zeolite-Portland cement mixture. J. Hazard. Mater..

[19] Wang Q., Wang D., Chen H. (2017). The role of fly ash microsphere in the microstructure and macroscopic properties of high-strength concrete. Cem. Concr. Compos..

[20] Wang L., Chen L., Cho D.W., Tsang D.C.W., Yang J., Hou D., Baek K., Kua H.W., Poon C.S. (2019). Novel synergy of Si-rich minerals and reactive MgO for stabilisation/solidification of contaminated sediment. J. Hazard. Mater..

[21] Chen L., Wang L., Cho D.W., Tsang D.C.W., Tong L., Zhou Y., Yang J., Hu Q., Poon C.S. (2019). Sustainable stabilization/solidification of municipal solid waste incinerator fly ash by incorporation of green materials. J. Clean. Prod..

[22] Noushini A., Castel A., Aldred J., Rawal A. (2020). Chloride diffusion resistance and chloride binding capacity of fly ash-based geopolymer concrete. Cem. Concr. Compos..

[23] Zhang H.Y., Qiu G.H., Kodur V., Yuan Z.S. (2020). Spalling behavior of metakaolin-fly ash based geopolymer concrete under elevated temperature exposure. Cem. Concr. Compos..

[24] Xie J., Wang J., Rao R., Wang C., Fang C. (2019). Effects of combined usage of GGBS and fly ash on workability and mechanical properties of alkali activated geopolymer concrete with recycled aggregate. Compos. Part B Eng..

[25] Nuaklong P., Jongvivatsakul P., Pothisiri T., Sata V., Chindaprasirt P. (2020). Influence of rice husk ash on mechanical properties and fire resistance of recycled aggregate high-calcium fly ash geopolymer concrete. J. Clean. Prod..

[26] Bajpai R., Choudhary K., Srivastava A., Sangwan K.S., Singh M. (2020). Environmental impact assessment of fly ash and silica fume based geopolymer concrete. J. Clean. Prod..

[27] Sandanayake M., Gunasekara C., Law D., Zhang G., Setunge S., Wanijuru D. (2020). Sustainable criterion selection framework for green building materials—An optimisation based study of fly-ash Geopolymer concrete. Sustain. Mater. Technol..

[28] Li N., Shi C., Zhang Z., Wang H., Liu Y. (2019). A review on mixture design methods for geopolymer concrete. Compos. Part B Eng..

[29] Tran T.T., Pham T.M., Hao H. (2019). Rectangular Stress-block Parameters for Fly-ash and Slag Based Geopolymer Concrete. Structures.

[30] Zhang P., Gao Z., Wang J., Guo J., Hu S., Ling Y. (2020). Properties of fresh and hardened fly ash/slag based geopolymer concrete: A review. J. Clean. Prod..

[31] Prachasaree W., Limkatanyu S., Hawa A., Sukontasukkul P., Chindaprasirt P. (2020). Manuscript title: Development of strength prediction models for fly ash based geopolymer concrete. J. Build. Eng..

[32] Zhang H., Li L., Sarker P.K., Long T., Shi X., Wang Q., Cai G. (2019). Investigating Various Factors Affecting the Long-Term Compressive Strength of Heat-Cured Fly Ash Geopolymer Concrete and the Use of Orthogonal Experimental Design Method. Int. J. Concr. Struct. Mater..

[33] Van Dao D., Ly H.B., Trinh S.H., Le T.T., Pham B.T. (2019). Artificial intelligence approaches for prediction of compressive strength of geopolymer concrete. Materials (Basel).

[34] Luhar S., Chaudhary S., Luhar I. (2019). Development of rubberized geopolymer concrete: Strength and durability studies. Constr. Build. Mater..

[35] Wang Y., Hu S., He Z. (2019). Mechanical and fracture properties of fly ash geopolymer concrete addictive with calcium aluminate cement. Materials (Basel).

[36] Javed M.F., Amin M.N., Shah M.I., Khan K., Iftikhar B., Farooq F., Aslam F., Alyousef R., Alabduljabbar H. (2020). Applications of gene expression programming and regression techniques for estimating compressive strength of bagasse ash based concrete. Crystals.

[37] Javed M.F., Farooq F., Memon S.A., Akbar A., Khan M.A., Aslam F., Alyousef R., Alabduljabbar H., Rehman S.K.U., Ur Rehman S.K. (2020). New Prediction Model for the Ultimate Axial Capacity of Concrete-Filled Steel Tubes: An Evolutionary Approach. Crystals.

[38] Özcan F. (2012). Gene expression programming based formulations for splitting tensile strength of concrete. Constr. Build. Mater..

[39] Tanyildizi H., Çevik A. (2010). Modeling mechanical performance of lightweight concrete containing silica fume exposed to high temperature using genetic programming. Constr. Build. Mater..

[40] Nour A.I., Güneyisi E.M. (2019). Prediction model on compressive strength of recycled aggregate concrete filled steel tube columns. Compos. Part B Eng..

[41] Iqbal M.F., Liu Q.F., Azim I., Zhu X., Yang J., Javed M.F., Rauf M. (2020). Prediction of mechanical properties of green concrete incorporating waste foundry sand based on gene expression programming. J. Hazard. Mater..

[42] Jalal M., Grasley Z., Gurganus C., Bullard J.W. (2020). Experimental investigation and comparative machine-learning prediction of strength behavior of optimized recycled rubber concrete. Constr. Build. Mater..

[43] Chou J.S., Pham A.D. (2013). Enhanced artificial intelligence for ensemble approach to predicting high performance concrete compressive strength. Constr. Build. Mater..

[44] Getahun M.A., Shitote S.M., Abiero Gariy Z.C. (2018). Artificial neural network based modelling approach for strength prediction of concrete incorporating agricultural and construction wastes. Constr. Build. Mater..

[45] Mashhadban H., Kutanaei S.S., Sayarinejad M.A. (2016). Prediction and modeling of mechanical properties in fiber reinforced self-compacting concrete using particle swarm optimization algorithm and artificial neural network. Constr. Build. Mater..

[46] Sebaaly H., Varma S., Maina J.W. (2018). Optimizing asphalt mix design process using artificial neural network and genetic algorithm. Constr. Build. Mater..

[47] Sudin R., Swamy N. (2006). Bamboo and wood fibre cement composites for sustainable infrastructure regeneration. Proc. J. Mater. Sci..

[48] Behnood A., Golafshani E.M. (2018). Predicting the compressive strength of silica fume concrete using hybrid artificial neural network with multi-objective grey wolves. J. Clean. Prod..

[49] Gandomi A.H., Babanajad S.K., Alavi A.H., Farnam Y. (2012). Novel approach to strength modeling of concrete under triaxial compression. J. Mater. Civ. Eng..

[50] Gandomi A.H., Yun G.J., Alavi A.H. (2013). An evolutionary approach for modeling of shear strength of RC deep beams. Mater. Struct. Constr..

[51] Ferreira C. (2006). Gene Expression Programming Mathematical Modeling by an Artificial Intelligence.

[52] Chen L., Kou C.H., Ma S.W. (2014). Prediction of slump flow of high-performance concrete via parallel hyper-cubic gene-expression programming. Eng. Appl. Artif. Intell..

[53] Beheshti Aval S.B., Ketabdari H., Asil Gharebaghi S. (2017). Estimating Shear Strength of Short Rectangular Reinforced Concrete Columns Using Nonlinear Regression and Gene Expression Programming. Structures.

[54] Kara I.F. (2013). Empirical modeling of shear strength of steel fiber reinforced concrete beams by gene expression programming. Neural Comput. Appl..

[55] Sadrossadat E., Ghorbani B., Hamooni M., Moradpoor Sheikhkanloo M.H. (2018). Numerical formulation of confined compressive strength and strain of circular reinforced concrete columns using gene expression programming approach. Struct. Concr..

[56] Nazari A., Riahi S. (2011). Computer-aided design of the effects of Fe2O3 nanoparticles on split tensile strength and water permeability of high strength concrete. Compos. Part B Eng..

[57] Gholampour A., Gandomi A.H., Ozbakkaloglu T. (2017). New formulations for mechanical properties of recycled aggregate concrete using gene expression programming. Constr. Build. Mater..

[58] Behnia D., Ahangari K., Noorzad A., Moeinossadat S.R. (2013). Predicting crest settlement in concrete face rockfill dams using adaptive neuro-fuzzy inference system and gene expression programming intelligent methods. J. Zhejiang Univ. Sci. A.

[59] Akbar A., Liew K.M., Farooq F., Khushnood R.A. (2020). Exploring mechanical performance of hybrid MWCNT and GNMP reinforced cementitious composites. Constr. Build. Mater..

[60] Ishak S., Lee H.S., Singh J.K., Ariffin M.A.M., Lim N.H.A.S., Yang H.M. (2019). Performance of fly ash geopolymer concrete incorporating bamboo ash at elevated temperature. Materials (Basel).

[61] Albitar M., Visintin P., Ali M., Drechsler M. (2015). Assessing Behaviour of Fresh and Hardened Geopolymer Concrete Mixed with Class-F Fly Ash. KSCE J. Civ. Eng..

[62] Alkroosh I.S., Sarker P.K. (2019). Prediction of the compressive strength of fly ash geopolymer concrete using gene expression programming. Comput. Concr..

[63] Hardjito D., Rangan B.V. (2005). Development and Properties of Low-Calcium Fly Ash-Based Geopolymer Concrete. https://www.researchgate.net/publication/228794879_Development_and_Properties_of_Low-calcium_Fly_Ash_Based_Geopolymer_Concrete.

[64] Joseph B., Mathew G. (2012). Influence of aggregate content on the behavior of fly ash based geopolymer concrete. Sci. Iran..

[65] Koza J.R., Poli R. (2005). Genetic Programming. Search Methodologies: Introductory Tutorials in Optimization and Decision Support Techniques.

[66] Saridemir M. (2010). Genetic programming approach for prediction of compressive strength of concretes containing rice husk ash. Constr. Build. Mater..

[67] Nath P., Sarker P.K. (2017). Flexural strength and elastic modulus of ambient-cured blended low-calcium fly ash geopolymer concrete. Constr. Build. Mater..

[68] Olivia M., Nikraz H. (2012). Properties of fly ash geopolymer concrete designed by Taguchi method. Mater. Des..

[69] Sarker P.K., Haque R., Ramgolam K.V. (2013). Fracture behaviour of heat cured fly ash based geopolymer concrete. Mater. Des..

[70] Long T., Shi X.S., Wang Q.Y., Li L. (2013). Mechanical properties and microstructure of fly ash based geopolymeric polymer recycled concrete. J. Sichuan Univ..

[71] Sujatha T., Kannapiran K., Nagan S. (2012). Strength assessment of heat cured geopolymer concrete slender column. Asian J. Civ. Eng..

[72] Vora P.R., Dave U.V. (2013). Parametric studies on compressive strength of geopolymer concrete. Proc. Procedia Eng..

[73] Wardhono A., Gunasekara C., Law D.W., Setunge S. (2017). Comparison of long term performance between alkali activated slag and fly ash geopolymer concretes. Constr. Build. Mater..

[74] Lokuge W., Wilson A., Gunasekara C., Law D.W., Setunge S. (2018). Design of fly ash geopolymer concrete mix proportions using Multivariate Adaptive Regression Spline model. Constr. Build. Mater..

[75] Mehta A., Siddique R. (2017). Properties of low-calcium fly ash based geopolymer concrete incorporating OPC as partial replacement of fly ash. Constr. Build. Mater..

[76] Ramujee K., Potharaju M. (2017). Mechanical Properties of Geopolymer Concrete Composites. Proceedings of the Materials Today: Proceedings.

[77] Sathanandam T., Awoyera P.O., Vijayan V., Sathishkumar K. (2017). Low carbon building: Experimental insight on the use of fly ash and glass fibre for making geopolymer concrete. Sustain. Environ. Res..

[78] Nuaklong P., Sata V., Chindaprasirt P. (2016). Influence of recycled aggregate on fly ash geopolymer concrete properties. J. Clean. Prod..

[79] Wongsa A., Zaetang Y., Sata V., Chindaprasirt P. (2016). Properties of lightweight fly ash geopolymer concrete containing bottom ash as aggregates. Constr. Build. Mater..

[80] Shaikh F.U.A. (2016). Mechanical and durability properties of fly ash geopolymer concrete containing recycled coarse aggregates. Int. J. Sustain. Built Environ..

[81] Shehab H.K., Eisa A.S., Wahba A.M. (2016). Mechanical properties of fly ash based geopolymer concrete with full and partial cement replacement. Constr. Build. Mater..

[82] Aliabdo A.A., Abd Elmoaty A.E.M., Salem H.A. (2016). Effect of cement addition, solution resting time and curing characteristics on fly ash based geopolymer concrete performance. Constr. Build. Mater..

[83] Okoye F.N., Durgaprasad J., Singh N.B. (2015). Mechanical properties of alkali activated flyash/Kaolin based geopolymer concrete. Constr. Build. Mater..

[84] Ganesan N., Abraham R., Deepa Raj S. (2015). Durability characteristics of steel fibre reinforced geopolymer concrete. Constr. Build. Mater..

[85] Assi L.N., Deaver E., Elbatanouny M.K., Ziehl P. (2016). Investigation of early compressive strength of fly ash-based geopolymer concrete. Constr. Build. Mater..

[86] Shaikh F.U.A., Vimonsatit V. (2015). Compressive strength of fly-ash-based geopolymer concrete at elevated temperatures. Fire Mater..

[87] Joseph B., Mathew G. (2015). Behaviour of Geopolymer Concrete Exposed To Elevated Temperatures School of Engineering. Ph.D Dissertation.

[88] Satpute S., Shirasath M., Hake S. Investigation of Alkaline Activators for Fly Ash-Based Geopolymer Concrete. http://ijariie.com/AdminUploadPdf/INVESTIGATION_OF_ALKALINE_ACTIVATORS_FOR_FLY_ASH_BASED_GEO_POLYMER_CONCRETE_ijariie3062.pdf.

[89] Lavanya G., Jegan J. (2015). Evaluation of relationship between split tensile strength and compressive strength for geopolymer concrete of varying grades and molarity. Int. J. Appl. Eng. Res..

[90] Nuruddin M.F., Demie S., Shafiq N. (2011). Effect of mix composition on workability and compressive strength of self-compacting geopolymer concrete. Can. J. Civ. Eng..

[91] Patankar S.V., Ghugal Y.M., Jamkar S.S. (2015). Mix Design of Fly Ash Based Geopolymer Concrete. Advances in Structural Engineering: Materials, Volume Three.

[92] Patankar S.V., Jamkar S.S., Ghugal Y.M. (2013). Effect of Water-To-Geopolymer Binder Ratio on the Production of Fly Ash Based Geopolymer Concrete. Int. J. Adv. Technol. Civ. Eng..

[93] Sumajouw M.D.J., Rangan B.V. (2006). Low-Calcium Fly Ash-Based Geopolymer Concrete: Reinforced Beams and Columns.

[94] Fareed Ahmed M., Fadhil Nuruddin M., Shafiq N. (2011). Compressive strength and workability characteristics of low-calcium fly ash-based self-compacting geopolymer concrete. World Acad. Sci. Eng. Technol..

[95] Deb P.S., Nath P., Sarker P.K. Properties of fly ash and slag blended geopolymer concrete cured at ambient temperature. Proceedings of the ISEC 2013 7th International Structural Engineering and Construction Conference: New Developments in Structural Engineering and Construction.

[96] Deb P.S., Sarker P.K., Nath P. Sulphate resistance of slag blended fly ash based geopolymer concrete Sulphate Resistance of Slag Blended Fly Ash Based Geopolymer Concrete. Proceedings of the 26th Biennial National Conference of the Concrete Institute of Australia. Concrete Institute of Australia.

[97] Galvin B., Lloyd N., Lecturer S. (1978). Fly Ash Based Geopolymer Concrete with Recycled Concrete Aggregate. Carbon N. Y. http://hdl.handle.net/20.500.11937/15785.

[98] Kusbiantoro A., Nuruddin M.F., Shafiq N., Qazi S.A. (2012). The effect of microwave incinerated rice husk ash on the compressive and bond strength of fly ash based geopolymer concrete. Constr. Build. Mater..

[99] Nuruddin M.F., Qazi S.A., Kusbiantoro A., Shafiq N. (2011). Utilisation of waste material in geopolymeric concrete. Proc. Inst. Civ. Eng. Constr. Mater..

[100] Hamad A.J. (2017). Size and shape effect of specimen on the compressive strength of HPLWFC reinforced with glass fibres. J. King Saud Univ. Eng. Sci..

[101] del Viso J.R., Carmona J.R., Ruiz G. (2008). Shape and size effects on the compressive strength of high-strength concrete. Cem. Concr. Res..

[102] Van Jaarsveld J.G.S., Van Deventer J.S.J., Lukey G.C. (2002). The effect of composition and temperature on the properties of fly ash- and kaolinite-based geopolymers. Chem. Eng. J..

[103] Gandomi A.H., Roke D.A. (2015). Assessment of artificial neural network and genetic programming as predictive tools. Adv. Eng. Softw..

[104] Babanajad S.K., Gandomi A.H., Alavi A.H. (2017). New prediction models for concrete ultimate strength under true-triaxial stress states: An evolutionary approach. Adv. Eng. Softw..

[105] Gandomi A.H., Alavi A.H., Mirzahosseini M.R., Nejad F.M. (2011). Nonlinear Genetic-Based Models for Prediction of Flow Number of Asphalt Mixtures. J. Mater. Civ. Eng..

[106] Aslam F., Farooq F., Amin M.N., Khan K., Waheed A., Akbar A., Javed M.F., Alyousef R., Alabdulijabbar H. (2020). Applications of Gene Expression Programming for Estimating Compressive Strength of High-Strength Concrete. Adv. Civ. Eng..

[107] Alavi A.H., Ameri M., Gandomi A.H., Mirzahosseini M.R. (2011). Formulation of flow number of asphalt mixes using a hybrid computational method. Constr. Build. Mater..

[108] Janardhanan T., Thaarrini J., Dhivya S. (2016). Comparative Study on the Production Cost of Geopolymer and Conventional Concretes. Int. J. Civ. Eng. Res..

[109] Lavanya G., Jegan J. (2015). Durability Study on High Calcium Fly Ash Based Geopolymer Concrete. Adv. Mater. Sci. Eng..

